# Vaccine Induced Herd Immunity for Control of Respiratory Syncytial Virus Disease in a Low-Income Country Setting

**DOI:** 10.1371/journal.pone.0138018

**Published:** 2015-09-21

**Authors:** Timothy M. Kinyanjui, Thomas A. House, Moses C. Kiti, Patricia A. Cane, David J. Nokes, Graham F. Medley

**Affiliations:** 1 School of Mathematics, University of Manchester, Manchester, M13 9PL, United Kingdom; 2 Department of Mathematics and WIDER, University of Warwick, Coventry, CV4 7AL, United Kingdom; 3 Kenya Medical Research Institute (KEMRI) – Wellcome Trust Research Programme, KEMRI Centre for Geographic Medicine Research – Coast, Kilifi, Kenya; 4 Public Health England, Salisbury, United Kingdom; 5 School of Life Sciences and WIDER, University of Warwick, Coventry, CV4 7AL, United Kingdom; 6 Department of Global Health and Development, London School of Hygiene and Tropical Medicine, London, WC1E 7HT, United Kingdom; National Institutes of Health, UNITED STATES

## Abstract

**Background:**

Respiratory syncytial virus (RSV) is globally ubiquitous, and infection during the first six months of life is a major risk for severe disease and hospital admission; consequently RSV is the most important viral cause of respiratory morbidity and mortality in young children. Development of vaccines for young infants is complicated by the presence of maternal antibodies and immunological immaturity, but vaccines targeted at older children avoid these problems. Vaccine development for young infants has been unsuccessful, but this is not the case for older children (> 6m). Would vaccinating older children have a significant public health impact? We developed a mathematical model to explore the benefits of a vaccine against RSV.

**Methods and Findings:**

We have used a deterministic age structured model capturing the key epidemiological characteristics of RSV and performed a statistical maximum-likelihood fit to age-specific hospitalization data from a developing country setting. To explore the effects of vaccination under different mixing assumptions, we included two versions of contact matrices: one from a social contact diary study, and the second a synthesised construction based on demographic data. Vaccination is assumed to elicit an immune response equivalent to primary infection. Our results show that immunisation of young children (5–10m) is likely to be a highly effective method of protection of infants (<6m) against hospitalisation. The majority benefit is derived from indirect protection (herd immunity). A full sensitivity and uncertainty analysis using Latin Hypercube Sampling of the parameter space shows that our results are robust to model structure and model parameters.

**Conclusions:**

This result suggests that vaccinating older infants and children against RSV can have a major public health benefit.

## Introduction

Respiratory syncytial virus (RSV) accounts for 66,000–199,000 deaths per year globally [1]. It causes a major burden of severe lower respiratory tract disease in children under 5yrs of age with an estimated 3,000,000 hospital episodes annually in all countries [1]. The vast majority of this burden occurs in low income countries [1]. The age distribution of childhood severe disease is highly skewed towards young infants (<6m), with around 50% of all RSV associated hospitalisations in this age group, attributed to small airway blockage through inflammation and sloughing of infected epithelial cells [2]. Early vaccine development, focused on this vulnerable group resulted in disaster when a formalin-inactivated preparation administered to naïve children led to exacerbated pneumonia and mortality upon natural RSV challenge [3]. Over the subsequent five decades, vaccination research for young infants has centred on live attenuated virus candidates (e.g. [4, 5]), but progress has been compromised by the presence of maternally derived antibodies (MAb), immunological immaturity, vaccine intolerance [3], and the legacy of uncertainty from the early vaccine failure.

Currently, there is a growth of interest in RSV vaccine development due to technical advances in delivery modalities, with around 45 candidates in various pre-clinical and clinical stages [6]. At the same time, there is recognition of the need to consider other groups to vaccinate to reduce the burden of infant disease. Older children aged 6 to 24 months are one of these groups—they have the advantage of a more mature immune system, lower levels of interfering maternal antibody and greater tolerance [7]. Live attenuated virus vaccines (LAV) administered intranasally to seronegative children of this age have been shown to be both immunogenic and well tolerated e.g. [4, 5]. Importantly they are also shown not to predispose the child to enhanced disease following wild type exposure, and, although the trials are small in size, indicated protective efficacy [8]. There is an active clinical trial program for LAV candidates for the prevention of RSV associated lower respiratory tract infection in young children [6, 9, 10].

The potential impact of vaccination of older children (including groups such as elder siblings) will depend upon the degree of direct and indirect protection due to the intervention. There are clear direct benefits to be gained from vaccinating older children since a significant proportion of RSV severe disease occurs beyond the first 6 months of life [11]. However, the main emphasis remains the prevention of disease in early infancy, which would have to accrue from indirect protection (often called herd immunity). Manufacturers and funding agencies would be encouraged if it were to be shown that vaccinating older infants offered an additional indirect protection to the young infant. To what extent older age group vaccination will confer herd immunity to the vulnerable young infant is unclear [12, 13]. This forms the subject of this paper.

Analysis of a cohort study in Kenya has demonstrated that the severe risk in young children is principally associated with age at infection, not their lack of experience of infection [14]. Studies of transmission in households have shown that at least half of transmission to infants is due to infection introduced to the household by their older siblings [15, 16]. Both of these results support the idea that immunisation of older children against RSV could be used to reduce disease in infants by delaying primary infection until they are older. Vaccination provides both direct protection to those who are successfully immunized with the vaccine and indirect protection for those who are not immunized by decreasing the number of the infectious individuals. Therefore, the impact of immunisation of children on infection and disease in infants is largely determined by the rate at which infection is transferred between different risk or age groups [17–19]. Consequently, we explore two different, data-based formulations of the age-related contact matrix, and also perform a parameter sensitivity analysis.

Our aim is to comprehensively explore the impact of childhood immunisation on RSV infection and morbidity, in the developing country setting, using mathematical predictive models paying particular attention to the pattern of mixing between age groups. What is the impact of vaccination of older infants and children on the public health burden of RSV in young infants?

## Methods

### Model

We have developed a deterministic model aimed at simulating the transmission dynamics of RSV in an age-structured population. The demographic structure subdivides the population into 99 age classes: 24 monthly age classes in the first two years of life and yearly age classes from the third year of life. Individuals older than 77 years have been put into the final age class. The selection of monthly age groups up to 2 years of age is chosen so as to capture the transmission dynamics and the impact of vaccination in the most critical age groups. The number of people in each age group is allowed to vary through a continuous ageing process and natural deaths.

**Fig 1:**
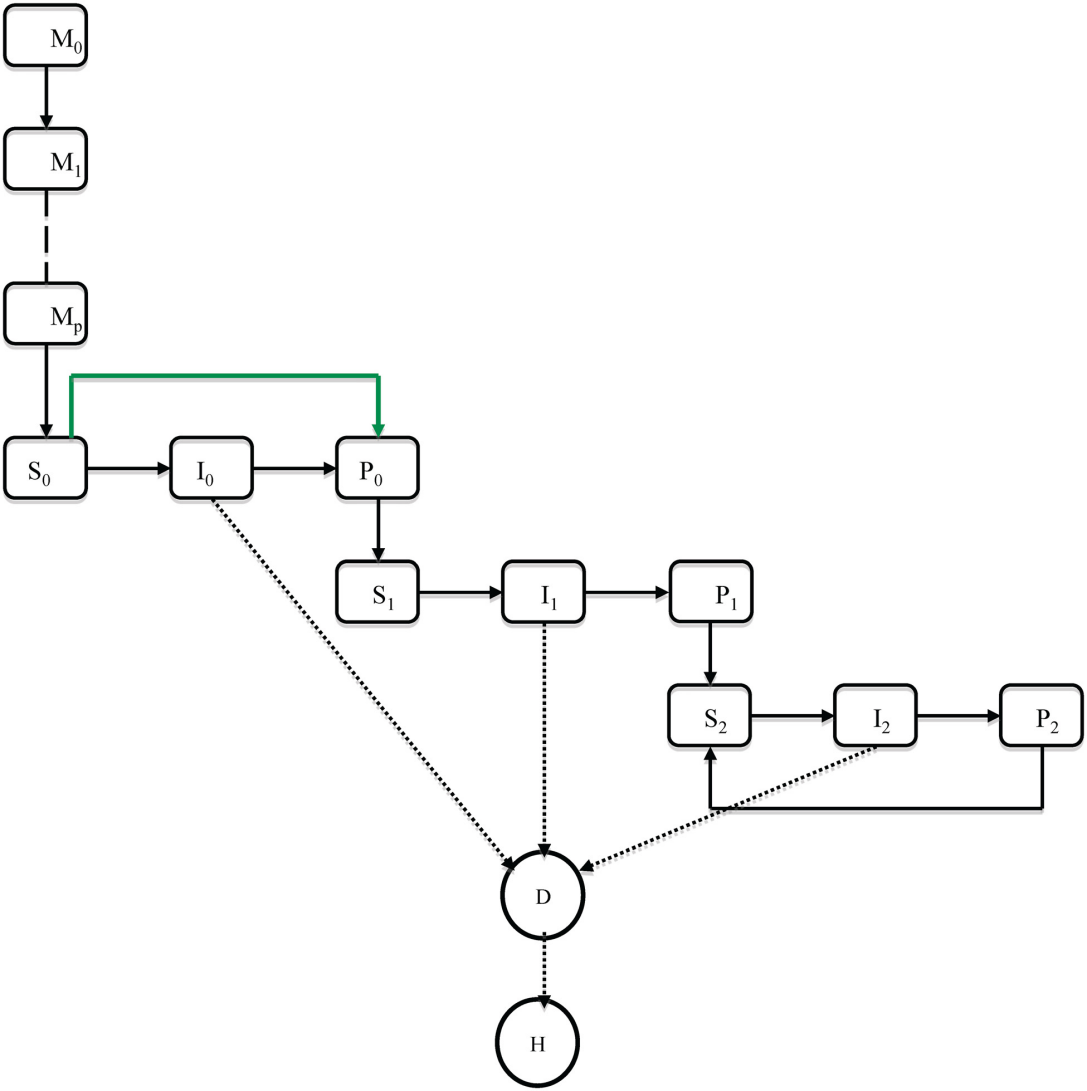
The model natural history framework. Rectangles represent mutually exclusive states, solid arrows are flows between states and dotted lines show classes that contribute to the rates of disease (D) and hospitalization (H). The solid green line shows the effect of vaccination, which prevents primary infection only and otherwise has an action like primary infection.

Individuals are modelled in 10 mutually exclusive groups as shown in Fig 1. Individuals are born with temporary but solid maternal immunity (*M*). We estimate the duration and distribution of protection by inclusion of a variable number of stages, *p*, within the *M* class, all of which confer full protection. After maternal immunity has waned, individuals enter a fully susceptible class (*S*_0_) and may experience primary infection *I*_0_. After each infection, there is a period of transient solid immunity (*P*_0_,*P*_1_,*P*_2_) before individuals become partially susceptible (*S*_1_,*S*_2_) reflecting that multiple infection episodes within epidemics are rare [20, 21]. Previously infected individuals have reduced susceptibility to infection [22], reduced duration of infection [23, 24] and reduced infectivity of infection on infection. The incidence of disease (*D*) is related to age and episode while hospitalisation (*H*) is related to age. The rates, with respect to both time and age at which individuals flow from one epidemiological state to another are described in the system of ODE shown in Eqn A in S1 File. The infectiousness of second and subsequent re-infections is parameterized relative to primary infected class. The per capita rate of infection experienced by individuals in age class *a* at time *t* is given by *λ*_*a*_(*t*). As the transmission of RSV is seasonal, we included a cosine function, fitting both the amplitude (i.e. strength of seasonality) and phase (i.e. timing). Table 1 gives a description of the parameters and their estimated values. The initial conditions for the state variables for each age class were taken to be the pre-vaccination numbers found by running the model for a period of 50 years to its stable limit cycle. This ensures that the transient population and infection dynamics effects are minimized during model fitting and vaccination. The set of ODE’s was solved numerically in Matlab^®^ [25] based on an explicit Runge-Kutta method of order (4,5) using an adaptive time step.

**Table 1:** Baseline parameter estimates used in the numerical simulations and the estimates of the fitted parameters.

Parameter	Description	Baseline value	Data source
*σ*_*k*_	Long-term immunity factor reducing the susceptibility of previously exposed individuals in *S*_1_ and *S*_2_	*σ*_1_ = 0.75 *σ*_2_ = 0.65	[22]
*ρ*_*k*_	Rate of waning of short-term immunity of recovered individuals	*ρ*_*0*_ = *ρ*_*1*_ = *ρ*_*2*_ = 2/*yr*	[20, 21]
*γ*_0_	Rate of recovery from primary infection, *I*_0_	40.6/*yr*	[15, 24]
*γ*_1_,*γ*_2_	Rate of recovery from secondary and subsequent infections: *I*_1_ *I*_2_	93.7/*yr*	[15, 23]
*α*_*k*_	Factor reducing infectiousness of *I*_1_ and *I*_2_	*α*_1_ = 0.5 *α*_2_ = 0.25	See text for justification
*N*	Population size	240,000	KHDSS**
*μ*_*α*_ *	Age-related death rate	Mid-year 2011 estimates from KHDSS** data	
*κ*_*α*_ *	Rate of ageing	0−≤24*m* = 12/*yr* 3−77*yrs* = 1/*yr*	Reciprocal of the length of the age class
*ω*	Duration of RSV specific maternal antibody protection	Fitted—see below	
*P*_*M*_	Number of classes of maternal protection	Fitted—see below	
*ξ*	Amplitude	Fitted—see below	
*ϕ*	Phase angle	Fitted—see below	
*q*	Infectivity parameter—Diary model	Fitted—see below	
*q*_*H*,_ *q*_*S*,_ *q*_*HS*_	Infectivity parameters—Synthetic model	Fitted—see below	
Estimated values of fitted parameters			
**Parameter**	**Diary model (95% CI)**	**Synthetic model (95% CI)**	
*ω*	2.3 (1.91 2.69) months	4.04 (3.6 4.5) months	
*P*_*M*_	1	1	
*ξ*	0.096 (0.088 0.105)	0.21 (0.19 0.22)	
*ϕ*	8.95e^-7^ (-0.0082 0.0083) (peak, 01 Jan)	2.216 (2.208 2.223) (peak, 26 March)	
*q*	0.00228 (0.00225 0.0023)	-	
*q*_*H*_	-	102.54 (98.65 106.43)	
*q*_*S*_	-	15,648 (15,633 15,663)	
*q*_*HS*_	-	2.56e^-12^ (-0.058 0.058)	

* The index *α* represents the age class i.e. *α* = 1,2,…,*n* where *n* = 99. *P*_*M*_ is the number of *M* sub-classes such that {*P*_*M*_: 1,2,…,*P*}. The rate of ageing from age class *α* is *κ*_*α*_. The different infection states are subscripted by *k* = 0,1,2.

**KHDSS—the Kilifi Health and Demographic Surveillance System [29]

Immunisation is assumed to confer protection to individuals such that it protects against primary infection. This assumption corresponds to the situation for a highly attenuated live virus vaccine that would not boost antibody titres in seropositive susceptibles [4]. Vaccination is implemented continuously and moves individuals from *S*_0_ to *P*_0_ (green line in Fig 1) as they pass a specified age gate. We consider a single efficacy parameter, coverage, which includes both the proportion vaccinated and the proportion protected by vaccination. To calculate the effect of direct protection, we run the model with a time invariant force of infection whose value is fixed at the pre-vaccination equilibrium.

### Data sources and model fitting

**Fig 2:**
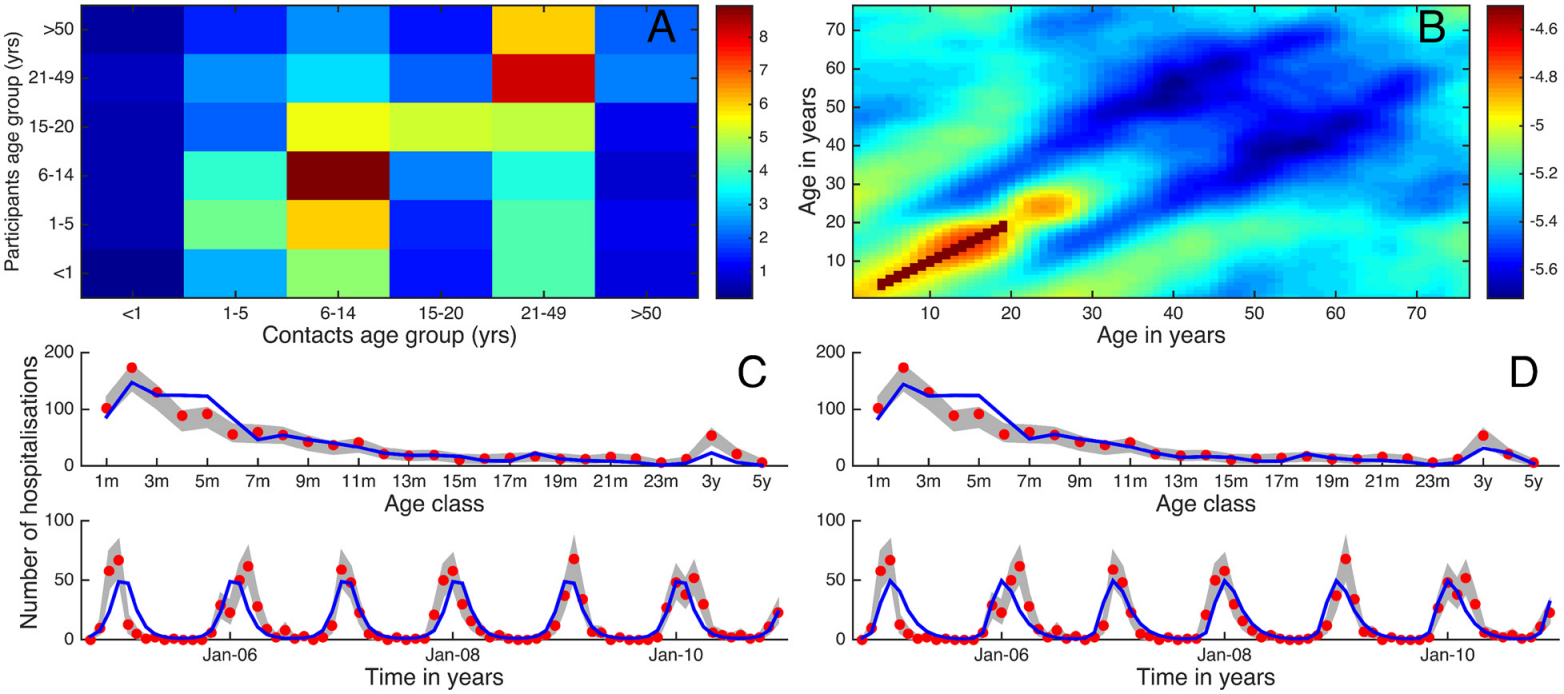
The estimated WAIFW matrices and the fits to hospitalisation data by age and time: left (A,C) are the diary WAIFW results, and right (B,D) are the synthetic WAIFW matrix. **A**) the mean number of contacts per day by participants (Y axis) by age group from a contact diary study in coastal Kenya corrected for population sizes. **B**) the fitted combination of three separate matrices for household structure, school attendance and homogeneous mixing plotted as the logarithm per person rate of contact (see S1 File). **C**) and **D**) show the age (top) and time (bottom) summed data (red dots), bootstrapped confidence intervals (grey bands) and the model fit (blue line) (note that the model was fitted to age-time data).

The transmission rates between individuals in the model are determined by age-related mixing: the who-acquires-infection-from-whom (WAIFW) matrix [26, 27]. Because the details of the age-related mixing are influential and largely unknown, we include two approaches to estimate WAIFW parameters. First, we use contact data that arise from diary data collected for a random sample of individuals who reside within the Kilifi Health and Demographic Surveillance System (KHDSS) [28, 29] (Fig 2A). Second, we estimate a synthetic contact matrix (Fig 2B) by weighted combination of three component matrices: a background homogeneous matrix, a matrix of contacts derived from household structure and a matrix of contacts derived from school attendance (Fig A in S1 File). The household occupancy data used for this synthetic matrix also arise from the registers of the KHDSS. Assuming that the WAIFW matrix is represented by *β*_ij_, and the contact matrix by *χ*_ij_, then we link the two by assuming that the self-reported age-specific number of social contacts are proportional to the age-specific number of potentially infectious contacts [30], i.e *β*_ij_ = *qχ*_ij_ where *q* is a disease specific infectivity parameter that we estimate by fitting the model to age-specific hospitalisation data.

The hospitalization data was obtained from Kilifi District Hospital (KDH), which is situated in the Kilifi County in Kenya, and serves as the primary care and referral facility for the predominantly rural population of the KHDSS [29]. The age specific fertility and mortality data used in the model were obtained from the KHDSS registers for the mid-year estimates for 2007. The model was fitted to RSV specific monthly admissions to KDH. Surveillance started in 2002 on all children <5 years admitted in KDH with clinical symptoms of pneumonia, either severe or very severe. Nasal specimens were collected as soon as possible after admission by nasal washing and screening for RSV antigen was undertaken using an Immunofluorescent Antibody Test (IFAT). The data used are the temporal and age-specific hospitalizations from Oct 2004 to Dec 2010. For more details on the study and the data, please refer to Nokes et al [11]. The risks of disease following infection are taken from a longitudinal cohort study of RSV infection in Kilifi, Kenya [14], and given in Table A in S1 File. The age-specific risk of hospitalization was estimated by fitting the static model (model with force of infection that is age-specific but constant in time) to hospitalization data. The initial vector was estimated from longitudinal data of RSV infections [12]. Once estimated from the static model, the risk of hospitalization remains fixed for fitting the dynamic model.

We calculate the expected incidence of hospitalisations from infections in two stages: the age-specific and infection-specific risk of disease given infection (*d*_0,*a*_,*d*_1,*a*_,*d*_2,*a*_), see Table A in S1 File, and the age-specific risk of hospitalisation given disease (*h*_*a*_), see Table B in S1 File. Eq 1 gives the expected number of hospitalisations from the model and this output is compared to the KDH hospitalization data during model fitting.

$$H(a,t)\;=\;h_{a}\lambda_{a}\overset{2}{\underset{i=0}{\sum }}d_{i,a}\sigma_{i,a}S_{i,a}$$ (1)

The model was fitted using statistical maximum-likelihood estimation assuming that the age and time counts follow a Poisson distribution. We optimized the model parameters by maximizing the log-likelihood as shown in Eq 2:

$$LL=\sum_{a\;=\;1}^{27}\{\sum_{t\;=\;1}^{T_{a}}\left(k(a,t)\text{log}H(a,t)-H(a,t)-\sum_{j=1}^{k(a,t)}\text{log}(j))\right\}$$ (2)

where *k*(*a*,*t*) is the expected incidence of hospitalizations at age *a* and time *t*, *T*_*a*_ is the number of time points at which the expected incidence data are made for each age *a* and *H*(*a*,*t*) is the corresponding expected incidences from the model at each age class *a* and time *t*. The negative log-likelihood was used as the objective function of *fmincon*, which is a minimization routine in the computational software Matlab^®^ [25]. To calculate the 95% confidence interval of the fitted parameters, we compute the central finite difference approximation to the Hessian of the log-likelihood estimates given the observed data to generate an asymptotic covariance matrix, and use a normal approximation [31]. To generate the bootstrapped confidence interval of the hospitalization data, we generated subsets, by both time and age, of data from the original set by sampling with replacement. The 95% confidence interval was taken as the region between the 97.5^th^ and the 2.5^th^ percentiles. The model output of interest is the proportion of RSV hospitalisations averted based on the long-term post-vaccination equilibrium when vaccination is implemented at a given age and coverage.

### Uncertainty and sensitivity analysis

Due to the structural complexity coupled with a high degree of uncertainty in some of the model input parameters, we performed global uncertainty and sensitivity analysis. Using Latin hypercube sampling (LHS) [32], we generated 200 sets of 10 parameters (see Table 2 for the parameters included, their probability density functions and the lower and upper bounds), for each of the WAIFW matrices, making a total of 400 different sets. For each of these sets, the fitted parameters are re-estimated from the age-specific hospitalisation data. We calculated the partial rank correlation coefficients for each of the input parameters and the model output.

**Table 2:** Model parameters that have been included in the sensitivity analysis, their upper and lower bounds and their probability density functions.

Parameter	Symbol	Lower and upper limits	Probability density function (pdf)
Duration of primary infection	*γ*_0_	[4–10] days	Triangular with peak at 9 day
Duration of second and subsequent infections	*γ*_1_,*γ*_2_	[1–5] days	Triangular with peak at 4 days
Duration of short term protection	*ρ*_0_,*ρ*_1_,*ρ*_2_	[2–17] months	Triangular with peak at 6 months
Reduction in susceptibility after first infection	*σ*_1_	[0–1]	Triangular with peak at 0.75
Reduction in susceptibility after second infection	*σ*_2_	[0–1]	Triangular with peak at 0.65
Reduction in infectiousness of secondary and tertiary infections	*α*_1_,*α*_2_	[0–1]	Uniform

### Ethical Review and consent

Kenya Ethics Review Committee (KEMRI/RES/7/3/1) and the Biomedical and Social Ethics Review Committee of the University of Warwick (134-07-2011) approved the study. Written informed consent was sought from participants aged ≥18 years and from parents or guardians of those aged <18 years. The data was analyzed anonymously to generate a Who Acquires Infection From Whom (WAIFW) matrix.

## Results

Fig 2 shows the two WAIFW matrices obtained from the contact diary data (A) and the synthetic contact matrix (B), respectively. Both indicate a strong within age group (assortative) mixing particularly in the school going age groups and secondary cross-generational mixing (e.g. parent to child; teacher to pupil), note the difference in the colour axis. The outcome of fitting the model is shown in Fig 2C and 2D. The directly estimated contact patterns and the synthetically estimated WAIFW give results indistinguishable in terms of fit to the hospitalisation data. The log-likelihood values for the diary and synthetic models were -1481.3895 and -1443.8595 with basic reproduction number (*R*_0_) of 7.08 and 25.60 respectively calculated as the dominant Eigen values of the WAIFW matrices [33]. Whilst the model reproduces the overall pattern, it is unable to predict the variation between different epidemics, which might be due to shifting viral genetics [34]. Similarly, the age groups within the mixing matrices do not match those in the data, which might explain the slight over-estimation in the 4–6m age groups.

**Fig 3:**
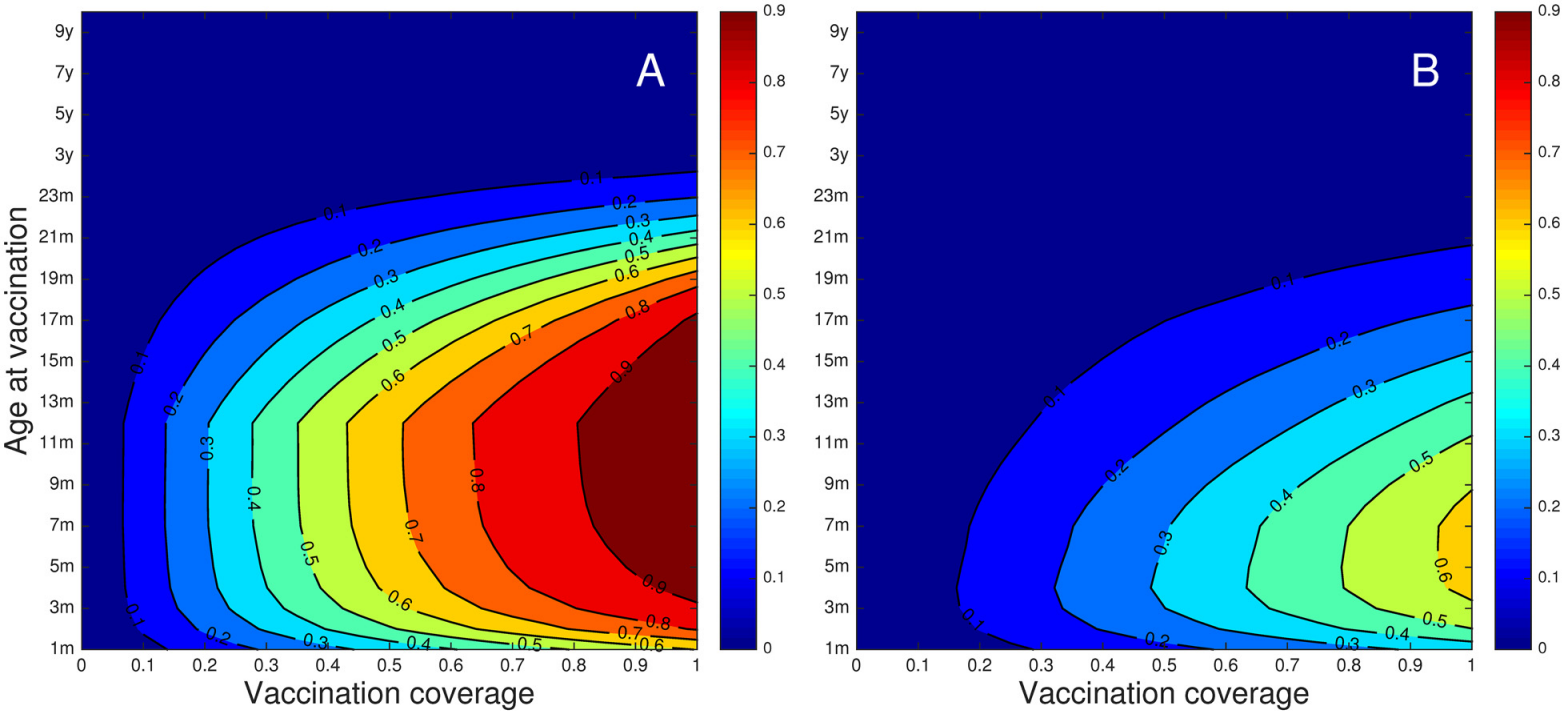
The effect of different ages at immunisation for different coverage with the base parameter set (Table 1) for the diary (A) and synthetic (B) contact matrices. The contour plots show the proportions of hospitalisations prevented by immunisation at different coverage (x-axis) by age at immunisation (y-axis) calculated over a 10 year time after transients have died away. **A**) diary WAIFW, estimated duration in M class, 2.3m. **B**) synthetic mixing WAIFW, estimated duration in M class 4.0m. In A, the most hospitalisations are prevented by vaccination at ~11m, and in B at ~6m.

**Fig 4:**
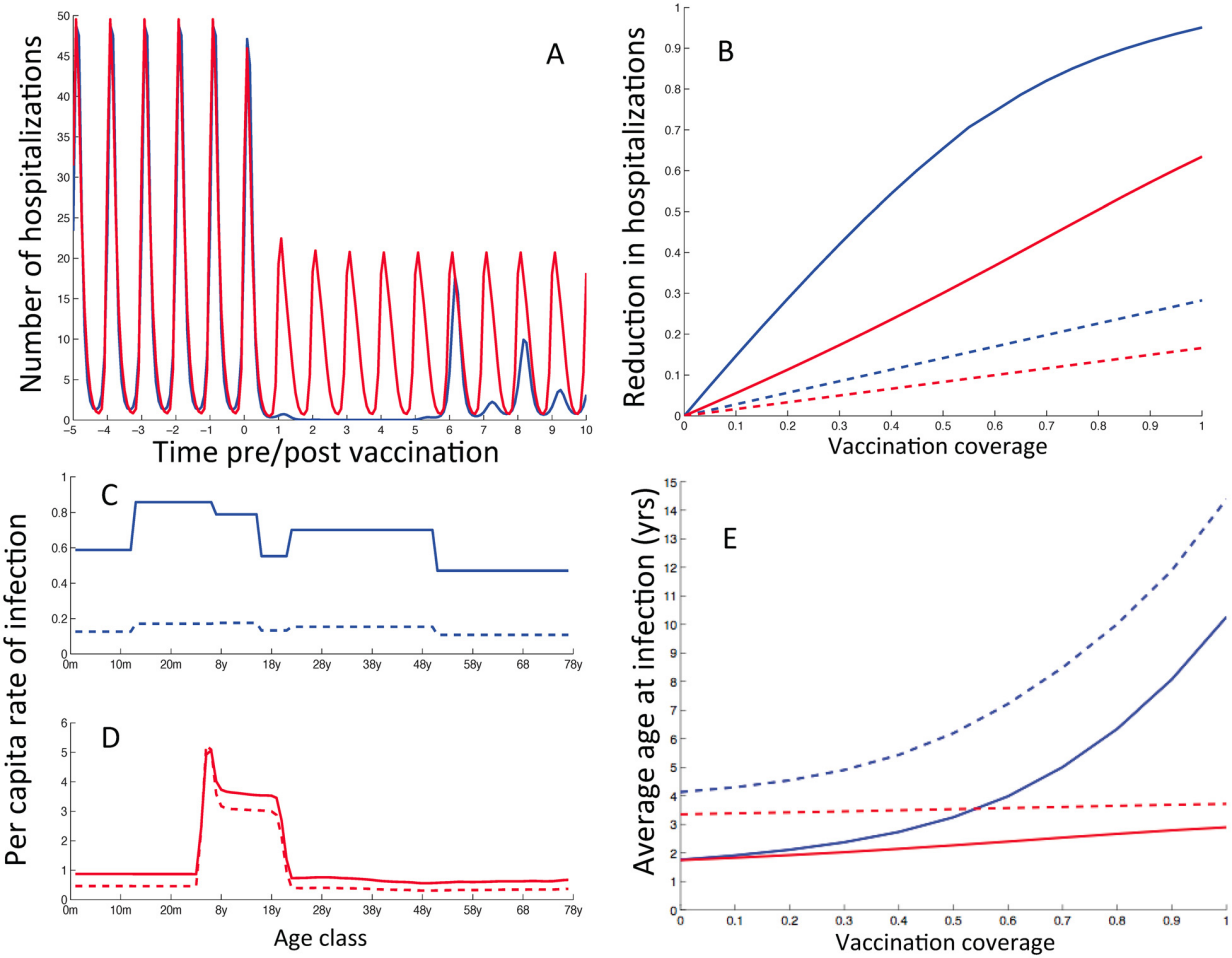
The impact of vaccination on infection and disease predicted using the diary (blue lines) and synthetic (red lines) WAIFW matrices and base parameter set. **A**) the short term temporal effect of vaccination pre and post immunization with vaccination implemented at time 0. **B**) the total impact (solid lines) and the direct effect (dashed lines) of vaccination with a vaccine given at 6 months for different coverages; blue and red lines the diary and synthetic model respectively. **C-D**) the age specific FOI at equilibrium pre-vaccination (solid) and with vaccination at 6 months at 70% coverage (dashed). **E**) the change in the average age at primary (solid line) and secondary (dashed line) with a vaccine given at 6 months of age.

Fig 3 shows the proportion of hospitalisations prevented, for different combinations of coverage (proportion immunised) and age of immunisation. Vaccination is potentially effective if given after maternal immunity is lost and before individuals have been infected for the first time. The predicted impact for the contact diary is higher, and maximum impact is more broadly distributed, resulting from a less peaked force of infection in infancy (see Fig 4C and 4D).

Fig 4A shows the short-term temporal effects of vaccination on the number of hospitalisations with immunisation implemented at time 0 at 6 months at 70% coverage. The diary model predicts a honeymoon period [35] of approximately 6 years before equilibrating to yearly epidemics with an alternating pattern of low and high peaks. On the other hand, the synthetic model does not predict a change in the pattern except that the epidemic peaks are reduced. Fig 4B shows that the total vaccine effect (solid lines) is much greater than the direct effect alone (dashed lines)–the difference is the indirect effect of protection of younger infants from infection. Fig 4C and 4D show the age-specific force of infection at equilibrium for the dairy and the synthetic models respectively. The solid lines shows pre-vaccination force of infection and the dashed lines show the force of infection with vaccination at 6 months at 70% coverage. It is clear from the figure that individuals aged between 2 and 20 years of age dominate the force of infection for the synthetic model. This can be attributed to the high number of contacts occurring within and between those age groups in the household. Immunisation is expected to change the average age at infection and this is shown in Fig 4E. This figure shows the change in the average age at primary (solid lines) and secondary (dashed lines) infections with a vaccine given at 6 months and for different vaccination coverages. The average age at primary infection increases from about 2 to 9 years (at 100% coverage) for the diary model (blue solid line) while the synthetic model predicts a relatively small increase, 2 to 2.5 years (red solid line).

**Fig 5:**
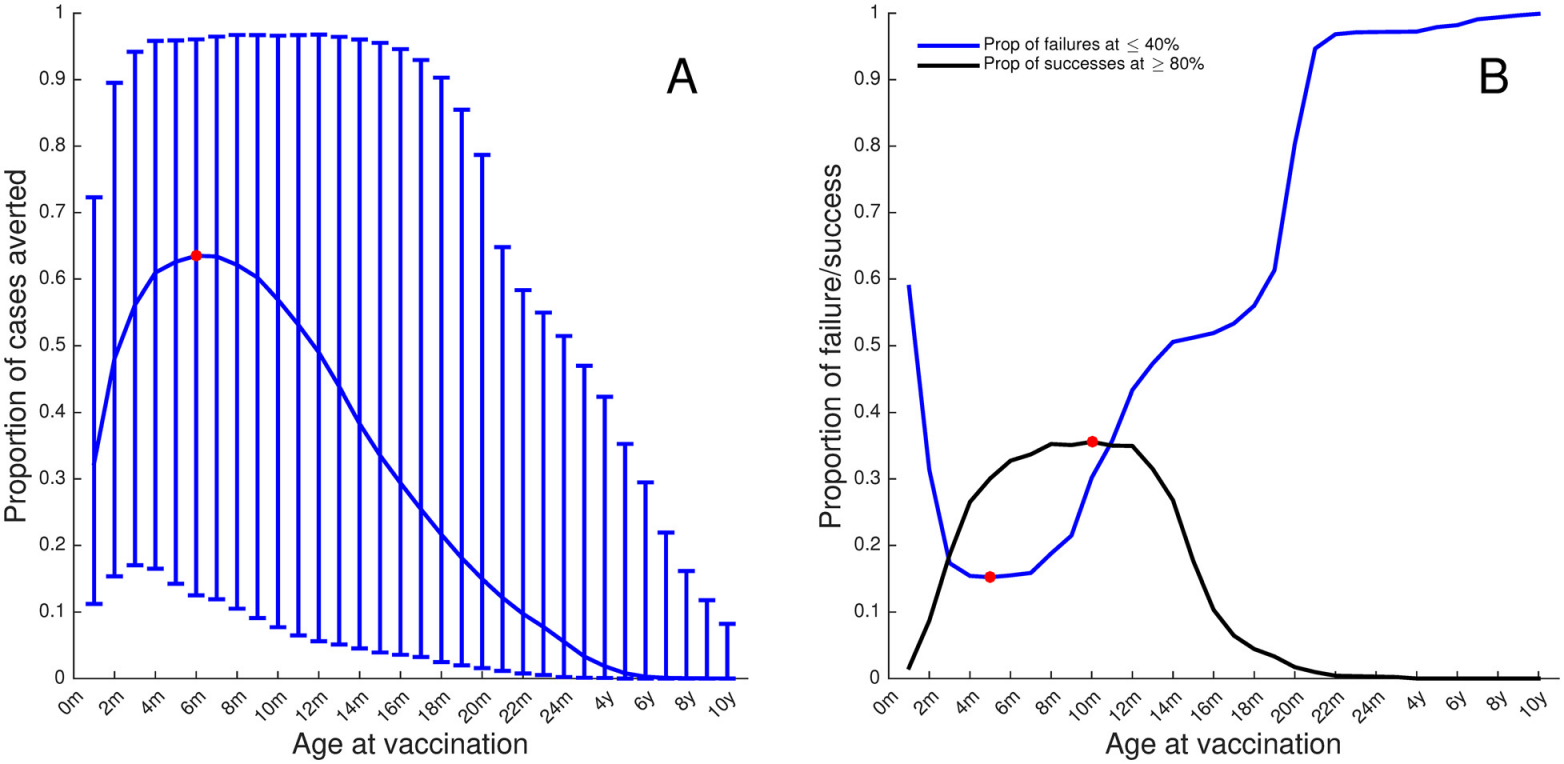
Sensitivity analyses. **A**: the median impact of vaccination in terms of proportion of RSV hospital admissions averted at 70% coverage at different ages with the bars showing the 95% range of results. The red dot indicates the highest median predicted impact (63% at 6m). **B**) the optimal age to vaccinate if the objective is to maximize the proportion of >80% reduction (black line) or minimize the proportion of <40% reduction (blue). The red dot shows the month at which the highest proportion of 80% reduction or the lowest proportion of 40% reduction is achieved on the black and blue lines respectively.

We then used LHS to explore the effects of uncertainty in estimating the values of the input variables on the prediction precision on the optimal age at vaccination, in particular exploring the range of potential optimum ages predicted. Fig 5A shows the proportion of RSV hospital cases averted at 70% coverage at different ages. There is considerable heterogeneity in the success of the vaccination programme depending on the assumed parameters: the bars show the range excluding the highest and lowest 2.5%, and the line the median. These results should not be interpreted as a probability distribution, but an assessment of the variation in optimum vaccination age given parameter uncertainty.

Fig 5B shows the effect of vaccinating at different ages (coverage fixed at 70%) with the parameter values used in the sensitivity analysis. We consider the proportion of parameter sets that maximize the probability of having more than 80% reduction in hospitalisations (black line) or ones than minimize the probability of having less than 40% reduction (blue line). The red dots shows that the highest proportion of 80% reduction is achieved with a vaccine given at age 10 months while the lowest proportion of a 40% reduction is achieved with a vaccine given at age 5 months. This result demonstrates that there is considerable variability in the potential effectiveness of a vaccination programme when both parameter and structural uncertainty are included, but that the age range of optimum outcome is relatively narrow.

## Discussion

We have presented an analysis of a mathematical model describing the transmission dynamics of RSV using data from a low-income country setting and explored both the long-term and the short-term impact of introducing RSV immunisation with two mixing assumptions. The outcome of fitting the model with different mixing assumptions gives results that are indistinguishable in terms of model fit and broadly consistent results in terms of the impact of immunisation. However, the results differ in a number of respects. First, there is a narrower range for the optimum age for the synthetic matrix, and the predicted impact is generally lower. Second, the two matrices are associated with very different underlying seasonal patterns of transmission. In fact the seasonal forcing that explains the observed epidemic pattern of RSV depends heavily on the details of the WAIFW. This is to be expected, and something worth exploring in more detail. If children are the “core group” for RSV as they are for measles [36], then presumably contact patterns are seasonally forced by the school year pattern, which is known, and would provide indirect information on the WAIFW matrix. Because the fitted dynamics of infection vary considerably depending on the WAIFW structure, but the optimum age window is unchanged, we suggest that this result will hold for most other epidemiological settings. Consideration of the impact of demographics and mixing in other settings remain to be explored.

From Fig 3, it is clear that vaccination is potentially effective if given after maternal immunity is lost and before individuals have been infected for the first time. This is a restatement of the ‘window problem’ first recognized for measles in high transmission settings [35], but with the extension that for RSV all settings are essentially high transmission because people are often reinfected [14, 15, 22, 37].

Further explanation for the impact of such immunisation is portrayed in Fig 4, which shows that immunisation works rapidly and effectively to reduce hospitalisation since severe disease is highly age-dependent (see Table A in S1 File). However, the dynamic patterns predicted for the mixing matrices are different. The WAIFW matrix derived from contact diaries predicts an initial dramatic decline in cases as virus circulation is almost stopped for the first few epidemic seasons. As with other infections, this results in a build-up of susceptibles and a subsequent rebound of epidemics [35]. Although virus circulation returns in older age groups, the immunisation programme prevents a return to pre-vaccination levels of disease. The WAIFW matrix derived synthetically allows virus transmission to be maintained and precludes a rebound effect. Given that viral circulation is not predicted to be greatly curtailed, there is very little possibility of adverse consequences of immunisation, for example caused by increased susceptibility in adults.

The two mixing matrices also predict two different mechanisms for vaccination effectiveness. Using the diary matrix, the model is essentially a susceptible-infected-resistant (SIR) framework in which primary cases drive the transmission and re-infections are “followers”, i.e. if the primary cases are reduced, then circulation is suppressed. Vaccination results in a dramatically increased average age at first infection (Fig 4E), effectively reducing the risk of infants becoming infected during the most susceptible ages. Using the synthetic matrix, reinfections are more important sources of transmission, so that transmission continues in the face of vaccination. Contact patterns are dominated by children (compare Fig 4C and 4D), so the indirect protection comes from preventing the young infecting the very young, i.e. it is more dependent on the exact contact pattern between the youngest age groups. Vaccination is predicted to delay, rather than prevent, primary infection, so that the increase in average age at primary infection is relatively small, but has a large impact. The estimate of viral transmissibility (as measured by the basic reproduction number) using the diary approach is below that using the synthetic matrix, and the two models (same structure, different parameters) fall either side of the re-infection threshold [37, 38]. For the diary matrix, RSV is unable to persist on secondary and tertiary infections alone, whereas the synthetic matrix model does not require primary infections to persist. Note the relative infectiousness of the different stages is fixed, but the overall infectiousness is estimated given the different mixing structures. This yields very different estimates for the basic reproduction numbers for the two models, even though they fit the data equally well prior to vaccination.

These observations make our vaccination results doubly robust: either primary cases are the drivers of RSV and transmission is greatly reduced, or primary cases are less important but vaccination reduces the source of transmission to the most vulnerable age groups. In either case we are predicting a substantial benefit from vaccination, with 50–70% of reduction in hospitalisation due to indirect protection.

The uncertainty and sensitivity results demonstrate that there is considerable variability in the potential effectiveness of a vaccination programme when both parameter and structural uncertainty are included. Given available information, the impact of 70% coverage could be to reduce hospitalisations by any value between 10% and 90%, although the best median values are >60%. However, the age of optimum immunisation is robust to these uncertainties. Whether the objective is to maximise the chances of a very successful intervention (>80% reduction in hospitalisation), reduce the chances of a poor intervention (<40% reduction) or maximise the expected outcome, the age window remains 5m to 10m.

The correlation between model outcomes and unknown parameters shows that the two models differ in their sensitivity. The diary matrix model emphasizes infection transmission between younger age groups (i.e. infants infected from primary infections), whereas the synthetic matrix emphasizes infection from older individuals (i.e. from secondary and subsequent infections to infants). Our focus has been on the impact of vaccination, but we have also demonstrated that the prediction of infection dynamics and consequent impact of immune responses to infection depends critically on the mixing assumptions. Recent results have also demonstrated the importance of demographic processes (especially birth rate) [39], and environmental (atmospheric) conditions [40] in determining RSV transmission dynamics. Recent individual-based models including explicit household and school-based transmission comes to a similar conclusion to our own [41]. Similarly, the impact of vaccination has been shown to be beneficial in much simpler models (e.g. [42]). Clearly, the impact of vaccination needs to be assessed in a wide variety of models including these and other processes such as viral genetics [34], but no model has yet suggested that immunisation against RSV would not be beneficial.

Immunisation is an extremely effective tool for preventing infection in individuals, but also results in protection of those not immunised through herd immunity. Careful consideration of the effects of herd immunity often changes the optimum allocation of vaccine [43–45]. Our results indicate that immunisation of young children (5–10m) is likely to be a highly effective method of prevention of severe RSV disease which arises predominately from infection in infants <6m old. The majority benefit is derived from herd immunity. We are greatly reassured that the different WAIFW, seasonality and reinfection threshold parameters can be combined to give the same endemic pattern and predict a similar public health benefit to vaccination.

Given the scarcity of contact data in different populations and the costly nature and difficulty of acquiring such data, we have developed a computational approach to derive the mixing pattern of the KHDSS population from household contacts combined with assumptions about school and out-of-school mixing pattern. This kind of approach has been independently developed for a number of European countries [46] with a notable agreement between the synthetic and the contact diary mixing data generated from the POLYMOD study [47]. The main advantage with this method is that it is general and can be easily used for regions without contact survey data as well as reconstruction of historical contact patterns for evaluation of the effect of demographic transition.

The vaccine modelled here only protects against primary infection. Importantly, this assumes that, as with primary infection, vaccination is followed by a transient period of protection and subsequent reduced susceptibility to reinfection and reduced infectiousness and duration upon reinfection. Although this is the most likely scenario for LAV since boosting of titres is absent in seropositives [4], reversion to a completely naïve susceptible state following waning vaccine immunity (or indeed natural infection immunity) cannot be ruled out [34]. Given that our results are dependent on reduction in disease rather than infection, a vaccine that reduces infectiousness of secondary and subsequent infections would have no less impact, but may be higher. Additionally, this work provides a conservative estimate of the impact of vaccination in assuming that the vaccine acts only on naïve individuals i.e. not previously infected. This would be the situation for a highly attenuated live virus vaccine that would not boost antibody in seropositive susceptibles.

We argue that RSV vaccine in 5–10m old children would result in significant herd immunity and lead to marked reduction in disease in those <6 months old. There are a group of vaccines that have already been subject to extensive trials and have relatively few obstacles remaining to develop an effective licensed product. We therefore propose that promotion of these candidates could have clear global public health benefit.

## Supporting Information

S1 File Supporting Information File 1. (DOCX)
