# Supplementary material for: Vaccine Induced Herd Immunity for Control of Respiratory Syncytial Virus Disease in a Low-Income Country Setting
Source: PLoS One. 2015 Sep 21;10(9):e0138018. doi: 10.1371/journal.pone.0138018 (PMC4577090; doi:10.1371/journal.pone.0138018)
Supplement: S1 File — (DOCX) [file pone.0138018.s001.docx]

Supporting Information

**Vaccine induced herd immunity for control of Respiratory Syncytial Virus disease in a low-income country setting – Supplementary Information**

Timothy M. Kinyanjui^1*^, Thomas A. House^1,2^, Moses C. Kiti^3^, Patricia A. Cane^4^, D. James Nokes ^3,5^, Graham F. Medley^6^

^1^School of Mathematics, University of Manchester, Manchester, M13 9PL, UK.

^2^Department of Mathematics and WIDER, University of Warwick, Coventry, CV4 7AL, UK.

^3^Kenya Medical Research Institute (KEMRI) – Wellcome Trust Research Programme, KEMRI Centre for Geographic Medicine Research – Coast, Kilifi, Kenya.

^4^Public Health England, Salisbury, UK.

^5^School of Life Sciences and WIDER, University of Warwick, Coventry, CV4 7AL, UK.

^6^Department of Global Health and Development, London School of Hygiene and Tropical Medicine, London, WC1E 7HT, UK.

Eqn A gives the model equations describing the rate with which individuals move from one epidemiological state to another as shown in Fig 1 in the main manuscript.

The indices $a$ and $j$ represent the age classes, $a,j=1,2,...,n$,$n=99$, $P_{M}$ is the number of $M$ sub-classes and $\iota=1,2,...,P_{M}$. The rate of aging from age class *a* is $\kappa_{a}$.

To assess the sensitivity of our results to different mixing patterns and to increase the robustness of our predictions of vaccination impact we investigated two contact matrices. As well as using the diary information, a synthetic contact matrix was estimated by combining a matrix of household contacts, and a matrix of school attendance [1]. We developed the synthetic contact matrix with the starting assumption that the WAIFW matrix is composed of three types of contacts: within household, within school and other contacts (homogeneous), each defined by a matrix as explained below. A contact is assumed to occur if two or more people share a physical environment.

**Household contacts**: A household is defined as an establishment where people share and eat from the same kitchen and all individuals within a household are assumed to contact all other individuals in the same household. To construct this matrix, we obtained household data (the number and age class of individuals living in every household) from the KHDSS population that was collected between September 2010 and January 2011. The matrix $T_{ij}^{H}$ is the total number of individuals (contacts) occurring within the household between age group $i$ and $j$. Since $T_{ij}^{H}$ accounts for reciprocity of contacts, then it relates to the mean number of contacts, $\Psi_{ij}^{H}$, as follows: $\Psi_{ij}^{H}=\frac{H_{ij}}{w_{i}w_{j}}$ where $w_{i}$ and $w_{j}$ are the population sizes in age classes $i$ and $j$ respectively. This resulting component is shown in Fig AA.

**School contacts**: In general, contacts within the school setting have been reported to occur within individuals predominantly of the same age range [2]. We have assumed that children will attend school between the age of 5 and 20 years. To populate this matrix, denoted by $\Psi_{ij}^{S}$, we equate the diagonal and the two parallel diagonals on either side of the main diagonal elements from age 5 to 20 years with the maximum value and half of the maximum value of the household matrix respectively. The resulting component is shown in Fig AB.

**General/other contacts**: A homogeneous contact matrix represents all the other contacts outside of the two settings determined above. The homogeneous mixing assumes that the mixing is similar across a wide age range. This matrix is denoted by $\Psi_{ij}^{HS}$.

To relate the three matrices, we linearly combine them to form the WAIFW matrix which we denote as $\beta_{ij}^{S}$ such that $\beta_{ij}^{S}=q_{H}\Psi_{ij}^{H}+q_{S}\Psi_{ij}^{S}+q_{HS}\Psi_{ij}^{HS}$ where $q_{H}$, $q_{S}$ and $q_{HS}$ are disease specific infectivity parameters for the household, school and general mixing respectively. The WAIFW matrix generated from the linear combination of the three components of mixing is shown in Fig 2B in the main manuscript.


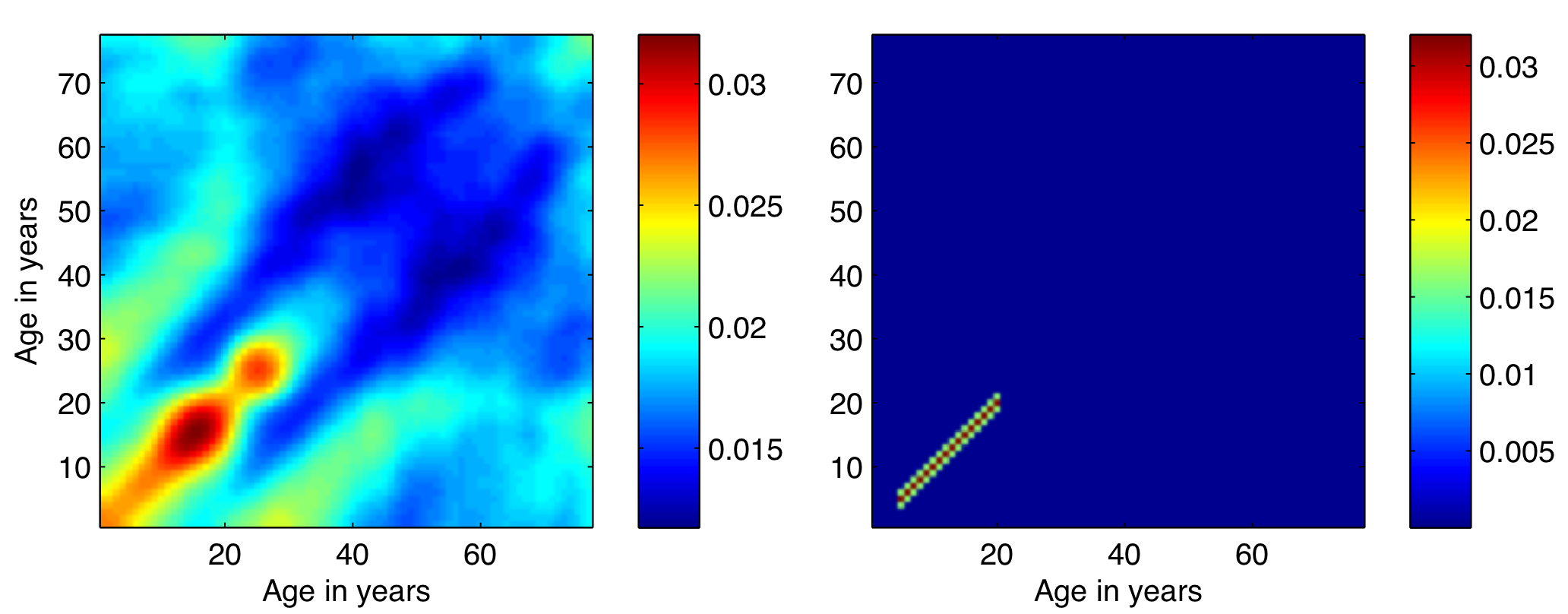


A

B

**Fig A.** The components of the synthetic mixing matrix. A is the pattern derived from the household occupancy data from KHDSS [has the same structure as POLYMOD]. B is the school and early work pattern. The third component (a matrix with the same values throughout) is not shown. These components are combined linearly to create the synthetic mixing matrix shown in Fig 2B in the main manuscript; see Table 1 in the main manuscript for fitted component weightings.

**Table A:** Baseline parameter estimates for the age-specific disease* risk following infection

| **Age (months)** | **Primary infection d_0_ (%)** | **Second and subsequent infections d_1_, d_2_ (%)** | **Source** |
| --- | --- | --- | --- |
| 0-2 | 31.2 | 5.0 | [3] |
| 3-5 | 28.6 | 5.0 |
| 6-8 | 20.0 | 5.0 |
| 9-11 | 13.0 | 5.0 |
| 12-17 | 7.6 | 5.0 |
| 18-23 | 2.0 | 2.0 |
| 24 + | 2.0 | 2.0 |

* Disease – Sever lower respiratory tract infection [3]

Table B: Parameter estimates for the age-specific risk of hospitalisation following disease

| **Age in months** | **Risk of hospitalisation (h) %** |
| --- | --- |
| 1 | 32.76 |
| 2 | 33.07 |
| 3 | 21.9 |
| 4 | 20.74 |
| 5 | 18.86 |
| 6 | 12.27 |
| 7 | 9.4 |
| 8 | 10.76 |
| 9 | 9.1 |
| 10 | 12.11 |
| 11 | 9.87 |
| 12 | 6.7 |
| 13 | 7.11 |
| 14 | 7.78 |
| 15 | 7.34 |
| 16 | 4.13 |
| 17 | 4.1 |
| 18 | 10.52 |
| 19 | 20 |
| 20 | 14.84 |
| 21 | 13.95 |
| 22 | 10.18 |
| 23 | 2.36 |
| 24 | 8.41 |
| 25-36 | 3.76 |
| 37-48 | 1.08 |
| 49-60 | 0.19 |

# The age specific risk of hospitalization was initially used from the paper published by Nokes et al [4]. However, given the problem of sample size in hospitalisations observed in children less than 5 years in the study, the estimates tended to give an over or under prediction of the model. Therefore, we calibrated the risk of age-specific hospitalizations folllowing disease by running a model with a time invariant force of infection to predict the age-specific profile of hospitalizations. The resulting optimised values are the ones we used in the model.

**Model fitting**

The model was fitted to RSV hospitalisation data from Kilifi District Hospital (KDH) using the maximum likelihood method. The fitted parameters are as shown in Table 1 (main text). They were allowed to vary within the domain of real numbers except for $P_{M}$. The parameter $P_{M}$ defines the number of maternally protected classes in the model. We fitted the rest of the parameters for each integer value of $P_{M}$ between 1 and 5, note that $P_{M}$ can only take integer values. We selected the model that gave the best fit, which had$P_{M}=1$; the log-likelihood values for the diary and synthetic models were -1481.3895 and -1443.8595 respectively.

# Uncertainty & Sensitivity analyses

Due to the model structural complexity and uncertainty in some of the model parameters, the behaviour of the model to changes in these parameters is investigated using Latin Hypercube (LHS) and Partial Rank Correlation Coefficient (PRCC) [5]. The estimation uncertainty for the parameters was investigated by specifying a probability density function (pdf) for each parameter and the variability in the pdf was used as a measure of uncertainty for each parameter. Table 2 (in the main text) shows the parameters that were subjected to the sensitivity analysis and their respective pdfs. A uniform pdf was defined for the factor reducing the infectivity of secondary and tertiary infecteds ($\alpha_{1},\alpha_{2}$) and therefore each interval in the pdf had an equal probability of being sampled. The upper and lower bounds of these parameters were assigned as [0 1] since that interval encompasses all the possible values. The pdf of the remaining parameters was taken to be triangular, reflecting the expectation that values close to the peak of the triangle are more likely compared to those at the boundaries. The upper and lower bounds were assigned based upon the available data, which have been reviewed in [6].

To sample the values for each parameter, the probability density functions were divided into 400 equi-probable intervals, 200 for each mixing assumption, from which we sampled without replacement [5,7]. For the diary model, each of the 200 samples were then paired with randomly chosen bootstrap samples of the diary contact matrix and then fitted to data. For the synthetic model, the samples were fitted to data and since the fitting process estimates 200 different synthetic WAIFW matrices, then there was no need to pair them with bootstrapped samples of the synthetic contact matrix. The output of interest from the 400 simulations was the optimal age at vaccination and the proportion of RSV hospitalization cases averted. Partial rank correlation coefficients were then calculated for each of the input parameters and the model output.

The principal difference between the diary and synthetic WAIFW matrix results is in the influence of parameter variation on the month of vaccination (Fig C and E). For both matrices the value of $\sigma_{2}$ is negatively correlated, i.e. if susceptibility after the second infection is higher then the optimum month of vaccination is lower, which reflects the narrowing of the susceptibility window. The optimum vaccination month is positively correlated in the diary WAIFW model with the duration of maternal protection and duration of primary infection, but the synthetic matrix results are not. The synthetic matrix results are negatively correlated with infectiousness of secondary and tertiary infectiousness and susceptibility to secondary infections, but the diary matrix results are not. These results suggest that the diary matrix emphasises infection transmission between younger age groups (i.e. from primary infections), whereas the synthetic matrix emphasises infection from older individuals (i.e. from secondary and subsequent infections).

Fig B: Shows the prediction uncertainty in the number of age specific (A) and time series hospitalizations (B) that is attributable to variation in the input parameters for the diary model. Subplot (C) shows the PRCC for each of the age classes and for each parameter as shown in the legend. Black circles imply that the PRCC of the parameter at that age class is statistically and significantly different from zero.

Fig C: Shows the PRCC for each parameter in the diary model influencing the optimal month at vaccination with a red scatter on the side of the bars showing parameters that are statistically different from zero.

Fig D: Shows the prediction uncertainty in the number of age specific (A) and time series hospitalizations (B) that is attributable to variation in the input parameters for the synthetic model. Subplot (C) shows the PRCC for each of the age classes and for each parameter as shown in the legend. Black circles imply that the PRCC of the parameter at that age class is statistically and significantly different from zero.

Fig E: Shows the PRCC for each parameter in the synthetic model influencing the optimal month at vaccination with a red scatter on the side of the bars showing parameters that are statistically different from zero.

References

1. Fumanelli L, Ajelli M, Manfredi P, Vespignani A, Merler S (2012) Inferring the Structure of Social Contacts from Demographic Data in the Analysis of Infectious Diseases Spread. PLoS Comput Biol 8: e1002673. Available: http://www.ncbi.nlm.nih.gov/pubmed/23028275. Accessed 3 October 2012.

2. Mikolajczyk RT, Akmatov MK, Rastin S, Kretzschmar M (2008) Social contacts of school children and the transmission of respiratory-spread pathogens. Epidemiol Infect 136: 813–822.

3. Ohuma EO, Okiro E a, Ochola R, Sande CJ, Cane P a, et al. (2012) The natural history of respiratory syncytial virus in a birth cohort: the influence of age and previous infection on reinfection and disease. Am J Epidemiol 176: 794–802. Available: http://www.pubmedcentral.nih.gov/articlerender.fcgi?artid=3481264&tool=pmcentrez&rendertype=abstract. Accessed 1 November 2012.

4. Nokes DJ, Okiro EA, Ngama M, Ochola R, White LJ, et al. (2008) Respiratory syncytial virus infection and disease in infants and young children observed from birth in Kilifi District, Kenya. Clin Infect Dis 46: 50–57.

5. McKay MD, Beckman R, Conover W (1979) A comparison of three methods for selecting values of input variables in the analysis of output from a computer code. Technometrics 21: 239–245. Available: http://www.jstor.org/stable/1268522. Accessed 6 April 2011.

6. Kinyanjui TM (2013) Modelling the Transmission Dynamics of RSV and the impact of Routine Vaccination The Open University.

7. Blower S, Dowlatabadi H (1994) Sensitivity and uncertainty analysis of complex models of disease transmission: an HIV model, as an example. Int Stat Rev Int Stat 62: 229–243. Available: http://www.jstor.org/stable/1403510. Accessed 4 April 2011.
